# New citation metrics released - Journal of Applied Oral Science

**DOI:** 10.1590/1678-7757-2020-ed001

**Published:** 2020-08-17

**Authors:** Karin Hermana NEPPELENBROEK, Linda WANG

**Affiliations:** 1 Journal of Applied Oral Science Brasil Editor-in-Chief, Journal of Applied Oral Science . Brasil; 2 Journal of Applied Oral Science Brasil Co-Editor-in-Chief, Journal of Applied Oral Science . Brasil

While the construction of reliable evidences has been addressing for robust investigations, it is of a great honor to shear the new impact factor for the Journal of Applied Oral Science (JAOS) in the Journal Citation Reports ^®^ (JCR) ^1^ of 1.797 based on the 2019 Science Edition. Out of the 91 journals indexed in this database in the Dentistry, Oral Surgery & Medicine category, JAOS is ranked at the 43 ^th^ place and the 2 ^nd^ quartile. Among all the Brazilian journals indexed in JCR, JAOS is the ١7 ^th^ out of 129 journals and presents the greatest impact factor of dental journals. Also according to other relevant metrics, JAOS reached increasing impact (2.9), as the CiteScore (CS) ^2^ from Elsevier, which calculates the indexes regarding Scopus database. In this metrics, we are ranked in the 48 ^th^ position among the 251 Dentistry journals, placed in the first quartile.

The announce of this news attest the strong team-spirit group, including the committed activities made by the Editors-in-chief, Associate Editors, and technical team, always unrestrained supported by the Institution since the former administrations. Since the journal is part of the digital library collection from the USP Journal Portal, supported by the philosophy of Open Access, JAOS manuscripts have reached nationwide and international coverage. This improvement was even more noticeable since 2012, when the USP Journal Portal became part of the network of scientific journal portals that use Open Journal Systems as a technological platform. Since its conception, as Journal of Applied Oral Science in 2003, the trajectory was towards to exclusively English-language manuscripts. This step was particularly crucial to achieve collaboration abroad, crossing national boundaries. In consequence, this Journal was a vehicle that could shear the knowledge built worldwide. To continue the improvements, the collaboration of the researchers as peer reviewers deserves a special recognition, as it corresponds to a volunteer action, in which their expertise, efforts and time-consuming to proceed all involved aspects to the decisions is of huge contribution. Technically, this well-connected work team optimized the management of the manuscripts to reduced turnaround time, specially in the first-round evaluation. Taking all these into consideration, it resulted in enhanced visibility which increased the demand of submissions. Therefore, the screening task has been crossing for more restrictive criteria, mainly based in the novelty and effective contribution. Other relevant markers in the latest years are the focus on providing a fast turnaround time for manuscripts and the continuous publication online modality adopted by the journal in 2018. Another major reason for JAOS’s success is the financial support provided over the last years by the USP Support Program for Periodical Scientific Publications, the National Council for Scientific and Technological Development (CNPq), and the Coordination for the Improvement of Higher Education Personnel (CAPES).

To accelerate the process and assure for recommended parameters, the editorial board reformulated the actions in different levels, mainly following the recommendations of SciELO (Scientific Electronic Library Online), an electronic library covering a selected collection of Brazilian scientific journals, which allows free access to full manuscripts. Due to the diligent work from the former editors-in-chief, since 2007, JAOS was indexed in database as SCOPUS, the world’s largest abstract and citation multidisciplinary database of peer-reviewed literature and web sources, comprehending the fields of Life Sciences, Health Sciences (including all publications indexed in Medline/PubMed electronic database), Physical and Social Sciences; Cochrane Library, an electronic database consisting of updated medical information sources based on evidence and systematic reviews; and Science Citation Index Expanded (SCIE), published by Thomson Scientific, which qualified JAOS as an international publication since very few dental journals are indexed in this database, thus demonstrating its highly selective inclusion criteria and procedures for acceptance in the collection, available through Web of Science ^®^ and the online version Sci Search ^®^ . In 2009, JAOS obtained the first impact factor in JCR (0.386), which resulted in significant changes as the implementation of an online submission and editorial system in 2007.

The news perspectives now are toward to advance the strategies related to the Open Science, a current scientific practice model to shear reliable and consistent data. Relevant changes will be made soon to provide more transparence of the process of the manuscripts, while to offer easy availability of them in shorter time, as the system of prerelease manuscripts, for instance. Digital platforms as Facebook, Twitter and Instagram also have been impulse the closer approach to the potential readers, including students and practitioners.

The best performance of JAOS in citation metrics produced a positive response, resulting in the submission of papers that meet the standards of quality as well as in the establishment of more selective criteria and assessment strategies, which apply both for authors and for reviewers. Lastly, this approach provided the required support and balance for the analysis of papers in the different areas of dental and speech-language pathology and audiology research. We look forward to continuing to receive excellent publications and high-quality reviews in fast turnaround time so that we have even more success rates in JAOS and its effective scientific contribution.
